# Inhibiting glucose-regulated protein 78 modulates lipid metabolism through controlling stearoyl-CoA desaturase 1

**DOI:** 10.1186/2049-3002-2-S1-P16

**Published:** 2014-05-28

**Authors:** Katherine Cook, Pamela Clarke, Anni Warri, Robert Clarke

**Affiliations:** 1 Department of Oncology and Lombardi Comprehensive Cancer Center, Georgetown University, Washington, DC, USA

## Background

Of the 230,000 new cases of invasive breast cancer diagnosed annually, 70% express the estrogen receptor-α and may be treated with anti-estrogen therapies. While these drugs induce clinical benefit, over 50% will develop resistance. Recently, we have determined a key role of the unfolded protein response endoplasmic reticulum protein chaperone, glucose-regulated protein 78 (GRP78), in mediating anti-estrogen resistance. We now expand the role of GRP78 to include regulation of lipid metabolism.

## Material and Methods

Liquid chromatography/mass spectroscopy matrix-assisted laser desorption/ionization-time of flight analysis, RNAi knockdown, and western blot hybridization was used to determine the metabolomic effect of GRP78 in anti-estrogen sensitive (LCC1) or resistant (LCC9) breast cancer cells. Furthermore, GRP78 targeting morpholinos were used to determine the effect of GRP78 inhibition *in vivo*.

## Results

Metabolic analysis indicated that inhibition of GRP78 through RNAi in breast cancer cells results in increased intracellular concentrations of polyunsaturated fats (PUFA) linoleate (18:2n6), linolenate [alpha or gamma; (18:3n3 or n6)], dihomo-linolenate (20:3n3 or n6), and dihomo-linoleate (20:2n6). GRP78 knockdown increased delta-6 desaturase (FADS2), possibly corresponding with the observed increase in PUFA. Moreover, knockdown of GRP78 decreased the endoplasmic reticulum protein stearoyl-CoA desaturase 1 (SCD1), further supporting a role for GRP78 in lipid metabolism. Knockdown of FADS2 or overexpression of SCD1 partially restored anti-estrogen resistance in GRP78 inhibited breast cancer cells, suggesting both proteins independently play a role in GRP78-mediated anti-estrogen resistance. Treatment of breast cancer cells with increasing doses of linoleic acid inhibited SCD1 and increased breast cancer cell death. Interestingly, combining anti-estrogens with linoleic acid had reduced the breast cancer cell-killing capacity than either treatment alone. However, anti-estrogens and linoleic acid treatment potentiated drug effectiveness in cells where GRP78 was inhibited, suggesting that PUFA regulation may be a secondary mechanism of anti-estrogen responsiveness dependent on GRP78 presence. Knockdown of SCD1 restored anti-estrogen sensitivity in LCC9 cells while overexpression of SCD1 in LCC1 cells promoted resistance. LCC9 orthotopic tumors treated with GRP78 targeting morpholino were re-sensitized to tamoxifen. Targeting GRP78 *in vivo* resulted in reduced SCD1 protein in GRP78 morpholino + tamoxifen treated tumors, demonstrating a key role of GRP78/SCD1 interaction in drug resistance.

## Conclusions

These data suggest that the effect of GRP78 knockdown on fatty acid metabolism and biogenesis may represent a new homeostatic ability of GRP78 to modify fatty acid metabolism. Moreover, the novel role of GRP78 controlling pro-tumorigenic SCD1 demonstrates the importance of targeting GRP78 in breast cancer treatment.

